# Screening the antifungal activity of essential oils against decay fungi from palmyrah leaf handicrafts

**DOI:** 10.1186/0717-6287-47-35

**Published:** 2014-08-15

**Authors:** Subajini Mahilrajan, Jeyarani Nandakumar, Robika Kailayalingam, Nilushiny Aloysius Manoharan, SriThayalan SriVijeindran

**Affiliations:** Palmyrah Research Institute, Kaithady, Jaffna, Sri Lanka; Department of Botany, University of Jaffna, Jaffna, Sri Lanka

**Keywords:** Essential oil, Growth inhibition, Minimal inhibitory concentration and Palmyrah leaf

## Abstract

**Background:**

The whitish tender leaves of Palmyrah are used for making handicrafts. The problem with these articles is discolouration with time and become more brittle due to fungal attack. This could be prevented by some protective coating. Instead of expensive and harmful chemicals we decided to test natural plant essential oils to control fungal attack. Palmyrah leaf article decay fungi were isolated from two different sites of Jaffna peninsula. In this investigation Antifungal Activity of different plant essential oils from neem *(Azadirachta indica*), castor (*Ricinus communis*), citronella (*Cymbopogon sp*) and camphor (*Cinnamomum camphora*) obtained from local market have been evaluated against isolated fungi. For screening of Antifungal activity, tests and controls were set to determine minimum inhibitory concentration (MIC) and Percentage of Growth Inhibition.

**Results:**

Morphologically three different types of Palmyrah leaf decay fungi were isolated and characterized as *Aspergillus niger, Aspergillus flavus* and *Penicillium sp*. Neem and castor oils have recorded no significant (0.05 > P) antifungal activity while citronella and camphor oils showed significantly different antifungal activity compared with control. Camphor oil and Citronella oil showed 100, 58.13% of average growth inhibition for *A. niger*. 96.38, 51.32% for *A.flavus* and 84.99, 72.76% for *Penicillium sp* respectively. Camphor oil showed highest percentage of growth inhibition at lowest minimum inhibitory concentration compared with citronella oil. Camphor oil was found to be highly antifungal and most effective against *A niger*, and *A. flavus*, compared with *Penicillium sp* and gave 100 percentage of growth inhibitions at 5, 1 and 15 ml/dl minimum inhibitory concentration respectively.

**Conclusion:**

Significantly higher broad-spectrum of antifungal activity was observed in camphor oil than other tested oils because it showed highest percentage of growth inhibition at lowest inhibitory concentration. Therefore it could be used for the development of new environmental friendly antifungal agent for the preservation of leafy handicrafts. Further formulation, field experiments are necessary to achieve this target.

## Background

*Borassus flabellifer* Linn (Palmyrah palm) these palm trees grow in a dry climate. The leaves of the palmyrah palm are thick, fibrous, initially strong and flexible, over time its flexibility decreases which is used to make handicrafts. They are also susceptible to insect attacks [1] and the lignified cells are particularly susceptible to degradation and discoloration. If not preserved properly they are subject to physical and fungal damage. Some of the most common deteriorating agents are climatic factors (e.g. variations in relative humidity and temperature) [1]. Agrawal [2] reported that conservation of palm leaf manuscripts using of citronella oil, camphor oil, or lemon grass oil on the surface of the leaf to keep it flexible. This prevents physical damage due to brittleness.

Essential oils are complex mixers comprising many single compounds. Chemically they are derived from terpenes and their oxygenated compounds. Each of these constituents has been shown to possess antibacterial, antifungal, antiviral insecticidal and antioxidant properties [3, 4]). The antimicrobial activity of different essential oils is known for many centuries. Large number of essential oils and their constituents were investigated for their antimicrobial properties against different bacteria and fungi strains [5, 6]. 

Among the essential oils citronella oil has shown inhibitory effect on biodegrading and storage of contaminating fungi [7].

Fungi are significant destroyers not only of foodstuffs, grains but also in leaves during storage, unfit for human consumption by retarding their nutritive value and often by producing mycotoxins [8, 9]. A sizeable portion of the world population living below poverty line in the developing and underdeveloped countries of Asia and Africa are suffering from health problems associated with consuming mycotoxin contaminated grains and cereals [10]. Even though effective and efficient control of air borne fungi can be achieved by the use of synthetic chemical fungicides; the same cannot be applied to leaf for reasons of pesticide toxicity [11–13] and durability of the leaf. Thus, there is a need to search for alternative approaches to store palmyrah leaf handicrafts without toxicity problems that are ecofriendly and cost effective.

## Results and discussion

### Isolation of palmyrah leaf article decay fungi

The palmyrah leaf is used for roofing, handicrafts and feed for livestock. The first two tender unexpanded whitish leaves and the next 12 young green leaves are used for making various handicrafts. The whitish tender leaves are used for making soft fine handicrafts while the young green leaves are used for making stronger, but coarse textured utility items like mats, baskets, packaging material, inner lining of heavy duty fibre baskets etc. The fungi affected palmyrah tender leaf articles were selected and using inoculation needle different colour colonies were inoculated into the PDA plate then incubated at room temperature for 4 days. After incubation black (B), green (G) and bluish green (A) colour of the colonies were observed. These three different colour colonies were selected for further study. Selected colonies were purified by repeated streaking. The purified fungi were maintained in agar slants at 4°C throughout the study and used as stock cultures.

### Characterization of isolated fungal strains

Selected fungi B, G & A were identified as *Aspergillus niger, Aspergillus flavus* and*Penicillium sp* respectively at species level in based on macroscopic and microscopic features (Table 1).

**Table 1:** **Macroscopic and microscopic features of isolated fungus**

Characteristics	B	G	A
Colony colour	Black with white reverse	Green with cream reverse	Bluish green with white revers
Hyphae	Septate hyaline	Septate hyaline	Septate hyaline
Conidial head	Radiate	Radiate	Radiate
Conidiophore	Smooth hyaline	Smooth hyaline	Simple
Vesicle	Globose	Subglobose	-
Conidia	Black globose	Globose	Round, Unicellular Unbranch
Phialide	Unisereate	Bisereate	

### Antifungal activity assay

**Figure 1:**
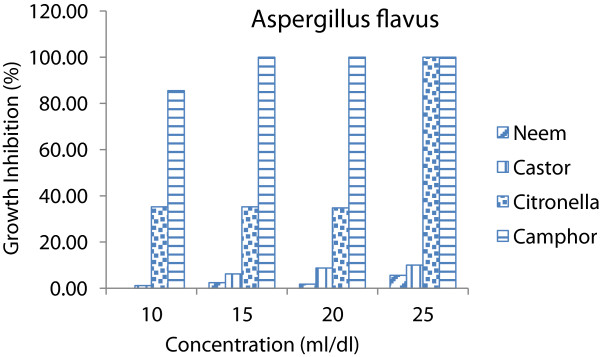
**Average growth inhibition of essential oils at different concentrations on** ***Aspergillus flavus.***

**Figure 2:**
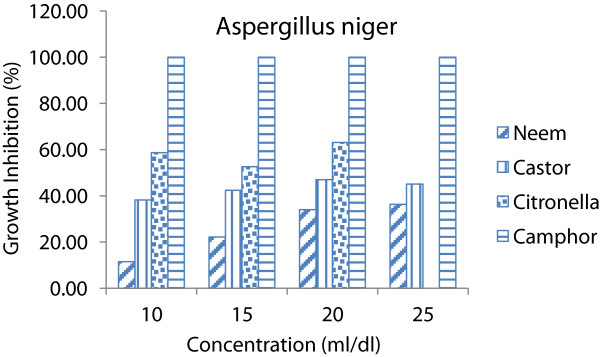
**Average growth inhibition of essential oils at different concentrations on** ***Aspergillus niger.***

**Figure 3:**
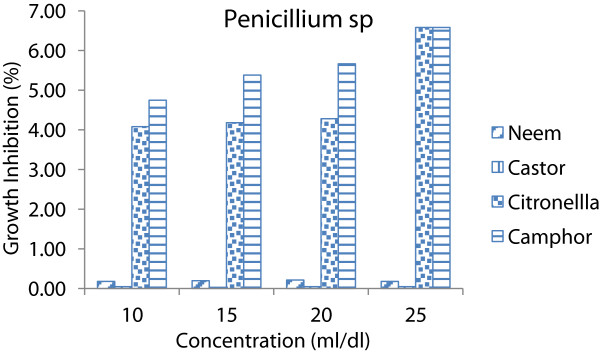
**Average growth inhibition of essential oils at different concentrations on** ***Penicilliumsp.***

**Figure 4:**
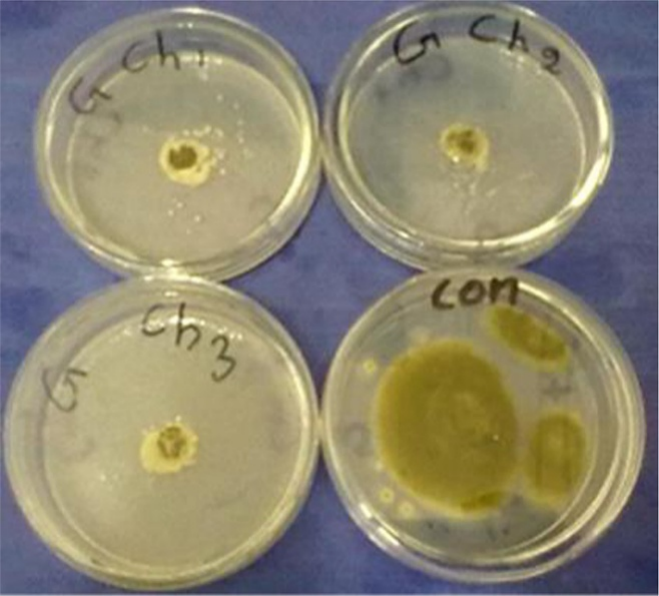
**Mycelial Growth inhibition of** ***A.flavus*** **at various concentration of camphor oil.** Upper left -5 ml/dl, Upper right - 10 ml/dl, Lower left -15 ml/dl and Lower right -control which not contained oil.

All species of fungi, using of any concentrations caused significant differences (p < 0.05) on inhibitory effect of essential oils. It could be seen that as essential oils concentrations increases the inhibitory effect increases (Figures 1, 2 and 3). In other words, the inhibitory effect of the essential oils was proportional to its concentration. This is in accordance with [14, 15]. Whereas increase in concentrations the susceptibility of fungi increases as well (Figure 4).

Until now, there were no reports on the effects of essential oils on Palmyrah leaf decay fungi. Based on our findings, camphor oil affected the growth inhibition of the studied fungi. In all studied fungi, the essential oils caused significant differences (p < 0.05) on growth inhibition rates. In other words, the effects of growth inhibitory of plant essential oils depend on species of fungi [15].

The results showed that two plant essential oil such as citronella and camphor oil caused 100% of growth inhibition on all species of fungi at 25 ml/dl concentration while neem and castor oil not caused 100% of GI at same concentration. Therefore lower concentrations (10, 15 and 20 ml/dl) of each essential oils were used to determine the MIC on these fungi (Table 2). Camphor oil was the most effective essential oil on the *A. niger, A. flavus and Peniillium* with growth inhibition average of 100, 96.38 and 84.99% respectively. Whereas the citronella oil showed minor effect on *A. niger, A. flavus and Peniillium* with growth inhibition average of 58.13, 51.32 and 72.76% respectively.

**Table 2:** **Growth inhibition (%) and MIC of essential oils**

Essential oils (v/v) (ml/dl)	Fungus					
	***Aspergillusniger***		***Aspergillusflavus***		***Penicilliumsp***	
	GI (%)	MIC	GI (%)	MIC	GI (%)	MIC
**Neem**						
10	0.00		11.52		2.74	
15	2.41		22.00		2.99	
20	1.77		34.02		3.24	
25	5.58		36.31		2.74	
**Castor**						
10	1.14		38.22		0.71	
15	6.21		42.41		0.46	
20	8.75		46.99		0.71	
25	10.01		45.08		0.71	
**Citronella**						
10	35.24		58.68		62.01	
15	35.24		52.62		63.53	
20	34.78		63.09		65.05	
25	100.00	*	NA		100.00	*
**Camphor**						
1	85.51		100.00	*	72.14	
05	100.00	*	100.00		81.76	
10	100.00				07	
15	100.00		100.00		100.00	*

*GI*: Growth inhibition (%), *MIC*: Minimum Inhibitory Concentra.

GI of *A. niger* was showed higher significant difference (p < 0.05) for citronella oil at 25 ml/dl and all the concentrations of camphor oil when compared with other oils similarly *A. flavus* showed higher significant difference (p < 0.05) for citronella oil at 25 ml/dl and camphor oil at 5, 10 and 15 ml/dl when compared with other oils while *Penicillium* showed higher significant difference (p < 0.05) only for 25 ml/dl of citronella and 15 ml/dl of camphor oil. *Penicillium*sp that isolated from palmyrah leaf was the most sensitive and most resistant to the studied essential oils.

Essential oils have two prominent features; low toxicity for people and environment due to their natural properties and low risk for resistance development by pathogenic microorganisms [16]. For these reasons and considering the results, we recommend the use of camphor oils for development of new and safe antifungal agent for the preservation of leafy handicrafts.

## Conclusion

The results showed that citronella and camphor oils were very effective on *Aspergillus niger,Aspergillus* f*lavus* and *Penicillium* sp. (palmyrah leaf article decay fungi)with growth inhibition average of 100% at 25 ml/dl concentration. Nevertheless, MIC of the essential oils was variable depending to species of fungi. *Penicillium sp.* was the most sensitive and most resistant to the camphor oil with 100% growth inhibition at 15 ml/dl concentration. Since growth inhibition of studied essential oils were evident in this study, they have potential to control of these palmyrah leaf article decay fungi and could be considered for developing new antifungal agent. Further field study need to be done to find out whether the use of this essential oil will prevent these fungal growth on leaf handicrafts after using this essential oil at cottage level handicraft industries in Sri Lanka.

## Methods

### Essential oils

The essential oils such as neem oil, castor oil, citronella oil and camphor oil, obtained from local market and exposed to UV radiation for 10 min then these oils were used for this study. These oils were selected based on literature survey and their use in preservation of leaf articles.

### Isolation of palmyrah leaf decay fungi collection of sample

Palmyrah handicrafts are usually affected by fungus during rainy season. Affected tender leaf articles of Palmyrah were collected from two design centers of Palmyrah Development Board during rainy season and used for the isolation of Palmyrah leaf article decay fungus.

### Preparation of potato dextrose agar media

#### PDA plates

Potato Dextrose Agar medium was prepared according to the manufacturer’s direction. After sterilization, the medium was allowed to cool to 50°C and poured in to sterile petridishes (20 mL/Petridish) under aseptic condition.

#### PDA slants

The PDA was prepared according to the manufacturer’s direction and 7 mL of the medium was poured into boiling tubes. The tubes were plugged with cotton wool and sterilized at 121°C and 15 lb/in^2^ for 15 min. The tubes were then cooled in an inclined position and used for storage of the fungus.

### Isolation of fungal strains

Fungus affected leaf handicrafts were brought to laboratory and using inoculation needle different colour colonies were streaked on sterile PDA plates in Zig-Zag manner and incubated at room temperature for 3- 4 days. Selected colonies were purified by repeated streaking and transferred to PDA slants and kept at 4°C.

### Characterization of isolated fungal strains

Selected fungal colonies were identified to species level based on macroscopic morphology and microscopic features.

### Antifungal activity assay

PDA medium with 1, 15, 20 and 25 (ml/dl) concentrations of the essential oils such as neem, castor & citronella and 1, 5, 10 & 15 (ml/dl) of concentrations camphor oil were prepared. About 15 mL of the medium was poured into each petridish, Tween-20 (Sigma) was incorporated into the agar medium to enhance oil solubility and allowed to solidify. Nine mm disc of 5 days old culture of the test fungi from the margin of the plates were placed at the center of the petridishes and incubated at room temperature for 4 days. After incubation the colony diameter was measured in millimeter. For each treatment three replicates were maintained. PDA medium without the essential oil served as control. Growth zones were measured at 4^th^ and 6 ^th^ days of incubation. The fungi toxicity of the oils in terms of percentage of growth inhibition of mycelia was calculated by using the formula:


Where dc = Average increase in mycelial growth in control,

dt = Average increase in mycelial growth in treatment [17].

The antifungal agent nystatin added to the agar plates (final concentration of 1.0 mg/l) served as a positive control for Aspergillus *niger, A. flavus* and *penicillium*_sp_.

#### Statistical analysis

MIC and percentage of inhibition were analysed by SAS package and the mean separation was done by LSD at p = 0.05.
